# Understanding Adsorption and Reactions at Aqueous Oxide Interfaces with Neural Network Potential Molecular Dynamics

**DOI:** 10.1021/accountsmr.6c00001

**Published:** 2026-05-14

**Authors:** Sanghyun J. Park, Abhinav S. Raman, Annabella Selloni

**Affiliations:** † Department of Chemistry, 6740 Princeton University, Princeton, New Jersey 08544, United States; ‡ Department of Chemical Engineering, Indian Institute of Technology Madras, Chennai 600036, India

## Abstract

Chemical processes at metal oxide–water interfaces are of central importance in geochemistry, biology, and energy technologies. A better understanding of these processes would allow us to make a significant step toward optimizing and controlling them, which could in turn lead to broader impacts. Computational modeling is indispensable to accomplishing this task because complexity and disorder often make it difficult to extract atomistic information from experiments. Balancing computational cost and accuracy, simulation schemes based on efficient machine learning representations of the potential energy surface (PES) predicted by ab initio calculations have become increasingly popular over the past decade. In particular, several studies have demonstrated the ability of machine learning models to accurately reproduce the complex ab initio PESs of aqueous oxide interfaces, allowing simulations of systems and processes that are not accessible with ab initio methods.

In this Account, we review our recent efforts to understand adsorption processes and reactions at aqueous oxide interfaces using deep potential molecular dynamics (DPMD), a simulation scheme employing deep neural networks (DNNs), which has proven to be quite successful in accurately describing many different systems in the condensed phase. After summarizing the DPMD methodology, we first review our work on the acid–base chemistry of oxide surfaces in contact with water, a fundamental characteristic that controls proton transfer and surface charge at the interface. We focus on the aqueous interface of rutile IrO_2_, an oxide material thus far considered the best catalyst for the oxygen evolution reaction (OER). We show that this interface is characterized by a large fraction of dissociated water and a strong Brønsted acidity of the surface sites, in good agreement with the experimentally measured value of the point of zero proton charge.

In our second example, we investigate how the adsorption of organic species from ambient air or water affects the structure and wettability of the aqueous interfaces of TiO_2_, a prototypical photocatalytic material. This is a question that is relevant to understanding the UV-induced hydrophilicity of TiO_2_ surfaces, a property at the basis of self-cleaning windows and related applications. Specifically focusing on formic and acetic acids, the two most common atmospheric organic acids, our simulations reveal that these acids control the wettability of TiO_2_ largely through acid–base chemistry at the interface rather than chemisorption on the oxide surface, a finding that could help improve the design of self-cleaning surfaces and photocatalytic devices.

Finally, we review our recent study of methanol at TiO_2_–water interfaces, a system whose interest is largely motivated by the role of methanol in enhancing photocatalytic hydrogen evolution on TiO_2_. Our simulations provide mechanistic insights into the coupled roles of the organic adsorbate and water at the TiO_2_ interface, with implications for how methanol enhances the activity of H_2_ evolution.

## Graphical abstract

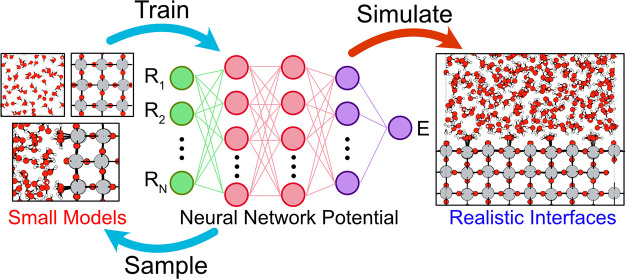

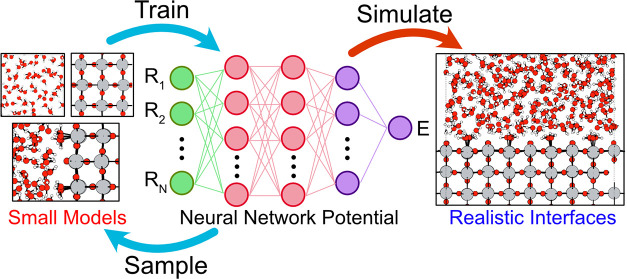

## 1 Introduction

The interfaces between metal oxides and water or aqueous solutions are central to many phenomena and applications, ranging from photo- and electrocatalytic water splitting to anticorrosion coatings and biomedical devices. While experimental surface-sensitive techniques are becoming increasingly available to investigate the structure and properties of these interfaces,^1–3^ the complexity of these systems often makes the analysis and microscopic interpretation of experimental results quite difficult. Computational modeling has proven to be essential for overcoming such difficulties. Traditionally, computational studies have modeled aqueous oxide interfaces employing both molecular dynamics with classical force fields and density functional theory (DFT) based ab initio molecular dynamics (AIMD) simulations.^4,5^ AIMD simulations predict, in general, more robust and transferable potential energy surfaces relative to empirical potentials and have made considerable contributions to the understanding of the structure and properties of oxide–water interfaces. However, due to their high computational cost, the typical size and time scales (a few hundred atoms and ∼100 ps, respectively) of AIMD simulations are often insufficient to characterize systems of the complexity of metal oxide–water interfaces. For example, for many years AIMD simulations could not clearly identify the dominant character, molecular vs dissociated, of adsorbed water at TiO_2_ interfaces.^6^

This situation has improved significantly over the past decade, thanks to the development of machine learning potentials (MLPs) capable of accurately and efficiently representing the ab initio potential energy surfaces of solid–water interfaces.^7–13^ Owing to their much lower computational cost in comparison to AIMD, MLP based simulations can extend over 10^3^–10^4^ longer time and length scales, thus allowing the determination of properties that are hardly accessible by AIMD, such as the equilibrium concentration of surface hydroxyls at oxide–water interfaces,^10,12^ a property that plays a critical role in the reactivity. MLP based simulations have also revealed the dynamical nature of the equilibrium structure at aqueous oxide interfaces, showing how interfacial water continuously dissociates, recombines, and exchanges protons with the surface and other neighboring species.^10,14^

In this Account, we summarize these computational developments and how they have been used to obtain molecular-scale insight into adsorption and reactions at the aqueous interfaces of catalytic metal oxide materials. After an overview of the methods (Section 2), in Section 3, we characterize the hydration structure and acid–base properties of rutile IrO_2_,^15^ one of the most efficient electrocatalysts for the oxygen evolution reaction (OER), whose surface chemistry was recently demonstrated to play an important role in the reaction.^16^ Due to their importance in photocatalysis, TiO_2_–water interfaces have been intensely studied using MLPs.^10–12,17,18^ In Section 4, we investigate how the adsorption of atmospheric organic acids affects the interfacial water structure and the wettability of TiO_2_ surfaces under ambient conditions.^19,20^ Finally, in Section 5, analysis of the effects induced by adsorbed methanol at TiO_2_–water interfaces reveals significant differences between anatase and rutile, with methanol enhancing water dissociation on anatase and suppressing it on rutile.^21^

## 2 Methodology

### 2.1 General Remarks

While different types of MLPs exist, in this Account we focus on deep neural network (DNN) potentials generated according to the deep potential molecular dynamics (DPMD) scheme,^8^ usually called deep potentials (DPs), which have been successfully applied to model many different solids, liquids, and interfaces. In the DPMD scheme, the potential energy *E* of each atomic configuration is written as a sum of “atomic energies” *E*_*i*_, i.e., *E* = ∑_*i* = 1_^*N*^ *E*_*i*_, where *E*_*i*_ is determined by the local environment of atom *i* within a smooth cutoff radius *R*_c_. *E*_*i*_ is constructed in two steps. First, for each atom a set of suitable structural descriptors is constructed, providing a representation of the local environment of that atom consistent with the required translational, rotational, and permutational invariance of the potential energy surface (PES) (see ref 22 for explicit examples). Next, this information is given as input for a DNN, which returns *E*_*i*_ as the output.^22^ The additive form of *E* naturally preserves the extensive character of the potential energy.

As with most MLPs typically used in atomistic simulations, the above-described DPs are intrinsically short-range. More recently, the DPMD scheme has been extended to generate DNN interatomic potentials that include long-range electrostatic interactions.^23^ The inclusion of such terms is essential for an accurate description of charge separation and transfer processes over distances larger than those captured by the short-range part of the potential.^24–26^ However, this is not the case for the systems described in this Account, for which the validity of the DPs was explicitly verified in the original studies.

Although obvious in principle, it is also worth stressing that the accuracy of DPMD simulations is ultimately determined by the quality of the DFT functional used for training the DP. Hence, the choice of an appropriate DFT functional is important. All the studies reviewed in this Account are based on the SCAN (strongly constrained and appropriately normed) meta-GGA functional,^27^ which is known to provide a good description of the properties of water and a large variety of molecules and materials.^28,29^

In the rest of this section, we present a brief general description of the training and validation of deep potentials. Details of these procedures for the specific systems reviewed in this Account are provided in the Supporting Information (SI).

### 2.2 Deep Potential (DP) Training

The training procedure used to generate a DP is schematically illustrated in Figure 1 for the example of the carboxylic acid-modified anatase TiO_2_(101)–water interface discussed in Section 4.^19^ In this procedure the neural network parameters are optimized to minimize total energy and force prediction errors by the DP model.^22^ The training always starts with the curation of an initial training data set of atomic configurations. For the example under consideration, this data set was composed both of configurations taken from the data set used to train previous DP models (bulk TiO_2_, the pristine TiO_2_–water interface, bulk water, and formic acid (FA) and acetic acid (AA) solutions) and of configurations obtained by taking snapshots from new AIMD simulations (FA and AA-covered TiO_2_–water interfaces at different coverages). DFT calculations on the selected configurations provided the energies and forces that served as input to train a “coarse” DP using the DeePMD-kit package.^22^ (Here, “coarse” DP indicates the initial DP obtained from a minimal training data set that provides an initial guess representation of the potential energy surface for further iterative refinement.)

**Figure 1:**
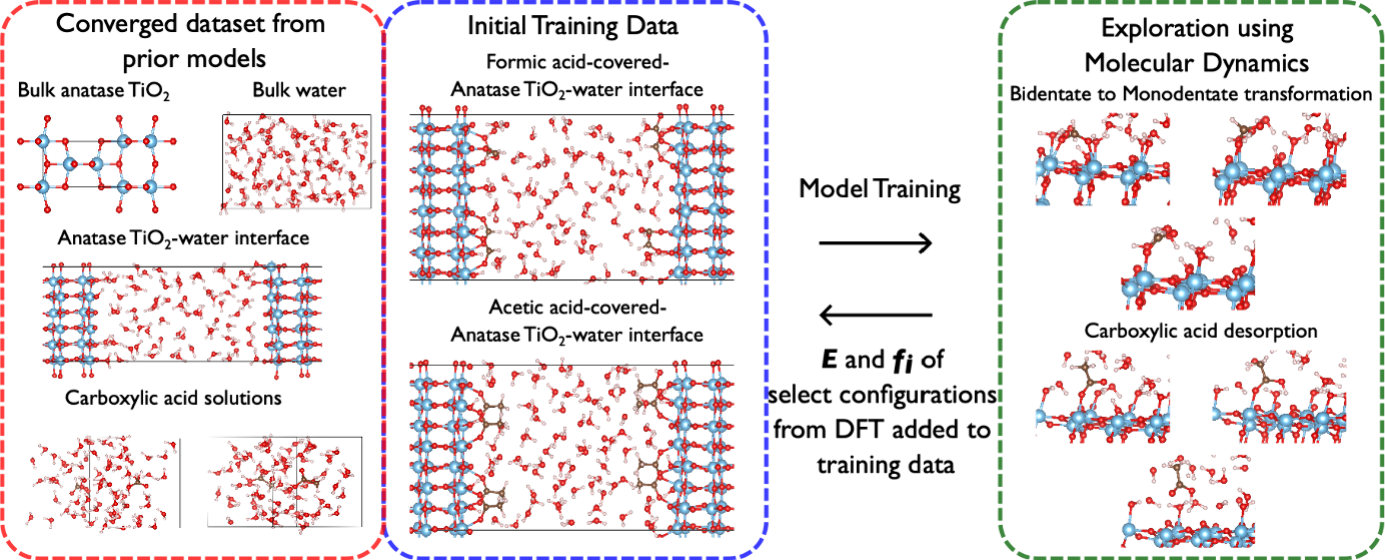
Schematic illustration of the active learning protocol used for the training of the DP for the specific case of the formic acid-covered anatase TiO_2_(101)–water interface. In the atomistic representations shown in the figure, the following color codes are used: blue, Ti; red, O; white, H; and brown, C. Reproduced with permission from ref 19. Copyright 2024 Elsevier B.V.

Using the trained coarse DP, an active learning protocol^9^ was then initiated. This involved the following: (a) exploration of the configuration space of the FA and AA-covered TiO_2_–water interfaces using short (∼100 ps) DPMD simulations with three DPs trained with different initialization random seeds;^22,30^ (b) evaluation of the maximum standard deviation of the atomic forces in the explored configurations between the three DPs; (c) DFT calculations to evaluate the atomic energy and forces for selected configurations that showed deviations above a predefined threshold (a maximum standard deviation in the range of ∼0.1 to 0.8 eV/Å is typically used); and (d) retraining of the DP with the included configurations added to the training data set. In the exploration step, configurations in a wide range of thermodynamic conditions were generated to help sample configurations involving the evolution of the interface structure. Additionally, for DPs that require accurate descriptions of dynamical processes such as dissociation reactions or surface desorption, it is necessary to incorporate explicit transition states sampled using enhanced sampling techniques.

The iterative active learning process was terminated, and the DP was considered converged when the average deviation in atomic forces between the three DPs was < 0.05 eV/Å over a 100 ps long DPMD simulation of each of the different interfaces of interest. The converged DPs were then used to perform nanosecond-time-scale DPMD simulations of the same systems. In general, all investigated properties were averaged over three independent simulations performed with three DPs trained with random initialization of their parameters. All DPMD simulations were performed using the LAMMPS package^31^ interfaced with DeePMD-kit.

### 2.3 Validation

Validating the ability of the trained DP to reproduce the DFT-predicted PES is the last essential step in the construction of a reliable DP. A first assessment of the accuracy of the DP is obtained by computing the root-mean-square (RMS) errors in the energy and the forces given by the DP relative to the corresponding values given by DFT, with the average taken over the configurations in all or a subset of the training set. More detailed information on the correlation between the training data and the model predictions is provided by the parity plots, *E*_DP_ vs *E*_DFT_ and *f*_DP_ vs f_DFT_, where *f* denotes the norm of the forces. Parity plots for each of the systems described in this Account are provided in the SI. Based on these plots, we can also analyze the distribution of the errors in the energy and forces. These distributions approximately follow a Gaussian and Lorentzian distribution, respectively, centered at zero and with spreads comparable to the RMS values mentioned above.^19,30^

We can further test the ability of the trained DP to reproduce the DFT energies and forces of configurations not provided in the training set. To this end, the trained DP is used to perform DPMD simulations from which uncorrelated snapshots are extracted. The DP-predicted energies and forces for these configurations were then compared with those given by direct DFT calculations. For a reliable DP, the resulting errors should be very similar to those made for configurations present in the training set. These comparisons can also be performed exclusively on configurations involving dynamic processes, such as proton transfer, to successfully validate the ability of the DP to accurately represent them (see Figure S2 in the SI). Lastly, DPs can be further validated for important dynamical processes by computing potentials of mean force using forces predicted by both DFT and DPs and evaluating the agreement in the free energy profiles.

## 3 Acid–Base Chemistry of the IrO_2_–Water Interface

Metal oxides are known to develop a surface charge that is dictated by their Brønsted acidity and the pH of the interfacing solution.^6,32^ This surface charging process determines surface speciation, ion adsorption, and chemical reactivity, thus playing a critical role in diverse fields ranging from geochemistry to electro- and photocatalysis. The fundamental quantity that determines the effective surface charge is the point of zero proton charge (pH_PZC_). For conditions where the pH of the interfacing solution is <pH_PZC_, the surface becomes positively charged by adsorbing protons from the solution. Conversely, when the pH of the interfacing solution is >pH_PZC_, the surface acquires a negative charge by releasing protons into the solution. The pH_PZC_ is a macroscopic experimentally measurable quantity that has been tabulated for several metal (hydr)­oxides over the years.^33^ It is determined by the acid dissociation constants (p*K*_a_s) of the different surface sites on the metal oxide that can accept or release protons. The p*K*_a_s themselves are inaccessible to current experimental techniques, which motivates the need for robust computational models that can estimate them.^32,34^ This also serves as a conduit that connects macroscopic to microscopic surface properties, which can be exploited to develop a molecular understanding of interfacial processes.

In this section, we summarize how DPMD simulations coupled with enhanced sampling methods^35^ have been used to understand the acid–base chemistry of the rutile IrO_2_–water interface, which plays a prominent role in electrocatalytic water splitting.^15^ To establish the theoretical framework for computing the pH_PZC_, we consider the rutile IrO_2_(110) surface, which is composed of two types of acid–base active sites in a 1:1 ratio. These are the 5-fold coordinated IrOH^–^/IrOH_2_ (cus) sites and the bridging O/OH^+^ (br) sites. The acid–base equilibria involving these two sites can be modeled according to the following proton transfer reactions:

1 $${\mathrm{OH}}_{\mathrm{br}}^{+}+H_{2}O\left(l\right)\to O_{\mathrm{br}}+H_{3}O^{+}\left(\mathrm{aq}\right)$$

2 $$H_{2}O_{\mathrm{cus}}+H_{2}O\left(l\right)\to {\mathrm{OH}}_{\mathrm{cus}}^{-}+H_{3}O^{+}\left(\mathrm{aq}\right)$$

The negative logarithm of the acid dissociation constants corresponding to eqs 1 and 2 gives p*K*_a,br_, and p*K*_a,cus_ respectively. Alternatively, eq 2 may also be written considering a completely hydroxylated surface as the reference, in which case the corresponding proton transfer reaction becomes

3 $${\mathrm{OH}}_{\mathrm{cus}}^{-}+H_{2}O\left(l\right)\to H_{2}O_{\mathrm{cus}}+{\mathrm{OH}}^{-}\left(\mathrm{aq}\right)$$

It is straightforward to obtain p*K*_b,cus_ from eq 3, which can be combined with the ionic product of water (p*K*_W_) to obtain p*K*_a,cus_ = p*K*_W_ – p*K*_b,cus_. As shown in ref 32, the pH_PZC_ is derived by adding the p*K*_a,br_ and p*K*_a,cus_ for eqs 1 and 2 under conditions of surface charge neutrality, i.e., [OH_br_^+^] = [OH_cus_^–^], and considering the 1:1 ratio of cus to br sites, viz.

4 $${\mathrm{pH}}_{\mathrm{PZC}}=\frac{{pK}_{a,\mathrm{br}}+{pK}_{a,\mathrm{cus}}}{2}$$

Moreover, the free energy for the dissociation of adsorbed water (Δ*F*_diss_) on the cus sites can also be obtained as^32^

5 $$\Delta F_{\mathrm{diss}}=2.303k_{B}T\left[{pK}_{a,\mathrm{cus}}-{pK}_{a,\mathrm{br}}\right]$$

where *T* is the temperature and *k*_B_ is Boltzmann’s constant. Thus, the pH_PZC_ and the individual p*K*_a_s of the surface sites directly control the speciation on oxide surfaces, where a lower Δ*F*_diss_ (larger dissociation fraction) would result from the increased basicity of the bridging sites.

From a computational standpoint, the crucial quantity that needs to be estimated is the free energy of proton transfer (Δ*F*) for eqs 1 and 2 (or eq 3), since p*K*_a_ = Δ*F*/2.303*k*_B_ *T*. Additionally, if eq 3 is used as a reference, in principle the p*K*_W_ should be estimated using the same methods to be fully consistent. One popular approach to estimate Δ*F* is the combination of AIMD with free energy perturbation (FEP) methods.^32,34,36,37^ Alternatively, enhanced sampling methods coupled with DPMD provide an estimate of Δ*F* as well as mechanistic insights into the proton transfer process.^15,25^

Using the latter approach, we estimated the free energies for the proton transfer reactions in eqs 1 and 3 considering the fully hydroxylated IrO_2_(110) surface as a reference. For simplicity, we took the experimental value of the p*K*_W_ (14.0) while also considering the sensitivity of the pH_PZC_ and Δ*F*_diss_ to changes of ±0.5 of this value (see Table S3 in the SI). We note that nuclear quantum effects (NQEs) are known to play an important role in proton transfer processes and in determining the absolute magnitudes of p*K*_a_s, as demonstrated for liquid water.^38^ While their inclusion is beyond the scope of this study, it warrants further detailed investigation. The resulting free energy surfaces (FESs) as a function of two collective variables (CVs) adapted from a previous study are shown in Figure 2.^39^ Specifically, the *s*_p_ CV represents the protonation state of the system, while *s*_d_ measures the distance between the pair of atoms that have exchanged protons. *s*_p_ changes from 0 to +1 for the deprotonation of the br site (OH_br_^+^ → H_3_O^+^; Figure 2a), resulting in a negatively charged surface. On the other hand, *s*_p_ goes from 0 to −1 for the protonation of the cus site (OH_cus_^–^ → H_2_O_cus_; Figure 2b), resulting in a net positive surface charge. Further details on the CVs and the convergence of the metadynamics simulations, verified via their diffusive behavior, are given in the SI.

**Figure 2:**
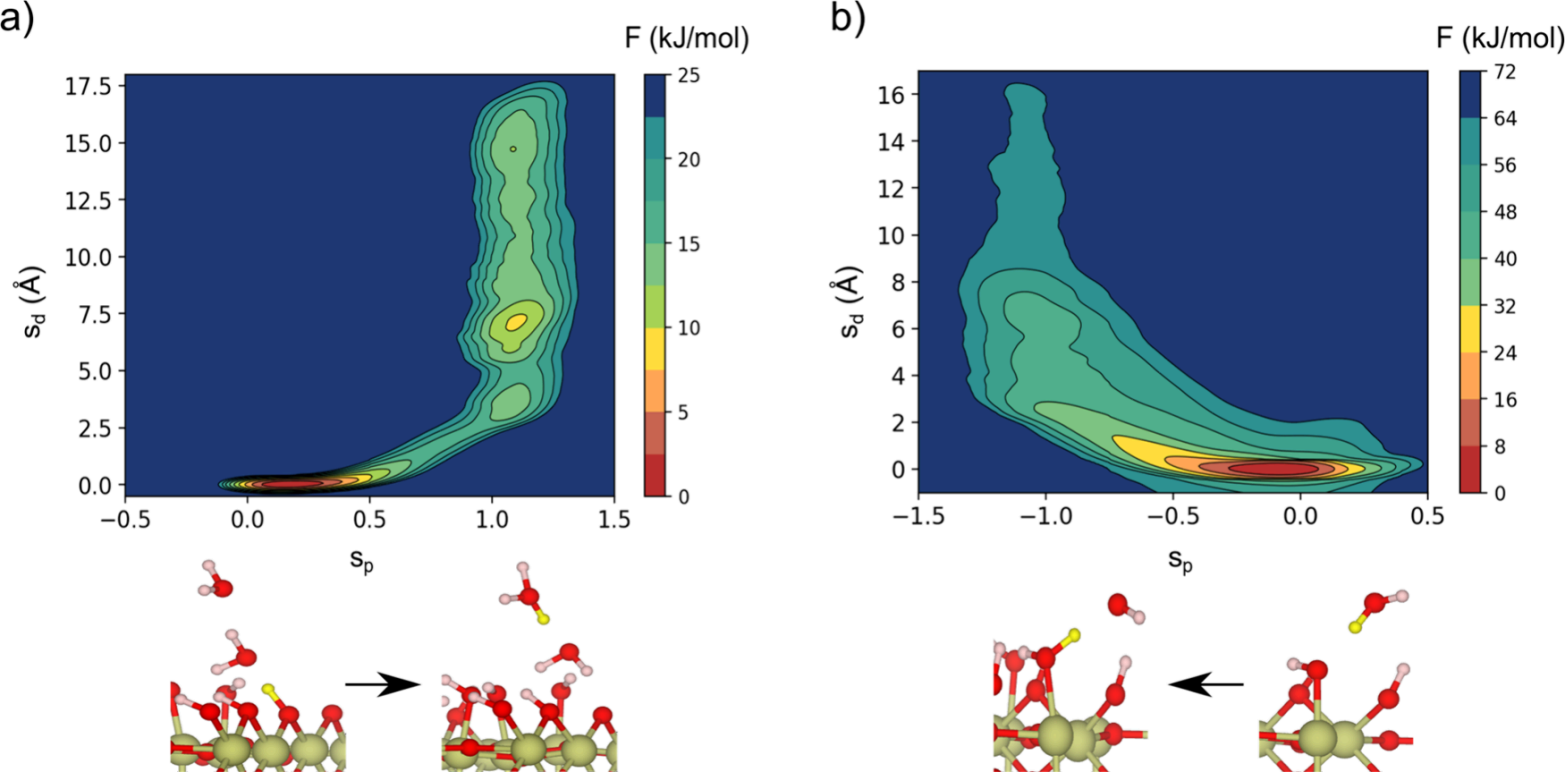
Free energy surface for (a) deprotonation of the OH_br_^+^ site (OH_br_^+^ → H_3_O^+^) and (b) protonation of the OH_cus_^–^ site (OH_cus_^–^ → H_2_O_cus_) from combined DPMD well-tempered metadynamics simulations^35,40^ of a (2 × 4) IrO_2_(110)–water interface. The inset shows an atomistic representation of the proton transfer process (color codes: gold, Ir; red, O; white, H; and yellow, transferring H^+^). Reproduced with permission from ref 15. Copyright 2023 American Chemical Society.

The corresponding Δ*F* and the p*K*_a_s can be straightforwardly obtained from the converged FES, and the pH_PZC_ was estimated as 3.4 ± 0.6, in excellent agreement with the experimental estimate of ∼3. This serves as a key parameter in the validation of the trained DP model to accurately represent the underlying potential energy surface. Further, the Δ*F*_diss_ was estimated to be −1.8 ± 4.9 kJ/mol, implying that water dissociation on the IrO_2_(110) surface is facile, an observation also obtained from long-time-scale (ns) equilibrium DPMD simulations of the interface that showed a water dissociation fraction of ∼83%.^15^ The exergonic dissociation free energy of H_2_O_cus_ obtained from the enhanced sampling simulations, as well as the dissociation mechanism of H_2_O_cus_ via proton transfer to the O_br_ sites and the rapid exchange of protons between H_2_O_cus_ and OH_cus_ observed in the equilibrium sampling simulations, suggests that the rate-determining formation of the OOH_cus_^*^ intermediate in the OER likely proceeds through 2O_cus_^*^ + H_2_O → OOH_cus_^*^ + OH_cus_^*^ or O_cus_^*^ + O_br_^*^ + H_2_O → OOH_cus_^*^ + OH_br_^*^. Both cases involve a “chemical transfer” of protons between different surface sites rather than into the solution, as observed in recent electrochemical experiments involving IrO_2_.^16^ Additionally, modulating the Brønsted acidity of selected surface sites to promote this proton transfer has been suggested as a tuning knob to alter the OER activity of other binary oxides.^41^

## 4 Adsorption of Organic Acids at TiO_2_–Water Interfaces and Their Effect on the Surface Hydrophobicity

Ever since the observed switchable wettability of TiO_2_ under UV light,^42^ efforts have been made to understand the hydrophobicity of TiO_2_ under ambient conditions. Over the years, and after many debates, it was largely agreed that the presence of atmospheric organic contaminants might play an important role,^43^ although the molecular picture remained unclear. More recently, experiments under controlled conditions reported the spontaneous formation of mixed formate-acetate monolayers on a rutile TiO_2_(110) surface that had previously been exposed to ambient atmosphere.^44,45^ The remarkable stability of these structures was attributed to the preferred bidentate (BD) adsorption configuration, where the carboxylic acid oxygen atoms bind to two undercoordinated (Ti_5c_) sites with the acid proton transferred to the adjacent bridging oxygen (O_2c_) atoms.^44,46^ However, the exact mechanism by which atmospheric carboxylic acids influence the wettability of TiO_2_ remains uncertain. This motivated us to use DPMD simulations coupled with enhanced sampling methods to reveal how formic acid (FA) and acetic acid (AA) control the wettability of both the anatase TiO_2_(101) (A101) and rutile TiO_2_(110) (R110) surfaces.^19,20^

To obtain insights into the structure of the interfacial water layers, we performed long-time-scale DPMD simulations (ns) of FA and AA-terminated A101 and R110–water interfaces, with the acids initially adsorbed in the stable BD adsorption configuration. Interestingly, we found that on the A101 surface several of the adsorbed formate and acetate species showed a spontaneous transformation from the BD to the monodentate (MD) configuration, leaving behind a vacant Ti_5c_ site, which was immediately occupied by a water molecule. This MD configuration appeared to be stable throughout the length of our simulation with the acids never returning to the BD configuration. On the other hand, the BD configuration showed remarkable stability for both acids on the R110 surface, emphasizing the distinct interfacial behaviors of the different TiO_2_ polymorphs.

Despite the spontaneous BD-to-MD transformation, the adsorbed acids did not desorb over the time scale of our equilibrium DPMD simulations, suggesting that the adsorption/desorption of FA and AA on/from the TiO_2_ surface in aqueous environments is an activated process with sufficiently large free energy barriers. To effectively sample the free energy surface (FES), we combined DPMD simulations with well-tempered metadynamics^35,40^ and collective variables (CVs) that adequately describe this process (details are given in the SI). Figure 3a,b shows the FES for the desorption of a single FA from the 0.5 monolayer (ML) FA-covered A101 and R110 interfaces, respectively. Two CVs, namely, the vertical distance of the carboxylic acid carbon from the Ti_5c_ sites (*D*) and the coordination number between the carboxylic acid oxygen and the Ti_5c_ sites (CN_O_carboxylic_–Ti_5c__), are used to represent the FES. The convergence of the metadynamics simulations was verified via the plateauing of the free energy difference between the fully desorbed and adsorbed states obtained over different simulation times (see Table S6 in the SI). The FES for both A101 and R110 are qualitatively identical, with the desorbed state being favored. In both cases, FA initially adsorbed in the BD configuration (CN_O_carboxylic_–Ti_5c__ ≈ 2) transforms to the MD configuration (CN_O_carboxylic_–Ti_5c__ ≈ 1), accompanied by the adsorption of water at the vacated Ti_5c_ site. This is followed by the desorption of FA as a formate ion (HCOO^–^), which is initially localized at the interface before getting hydrated in the bulk water region. The transformation from the MD to the desorbed state (CN_O_carboxylic_–Ti_5c__ ≈ 0) is once again accompanied by the adsorption of a water molecule on the newly vacated Ti_5c_ site.

**Figure 3:**
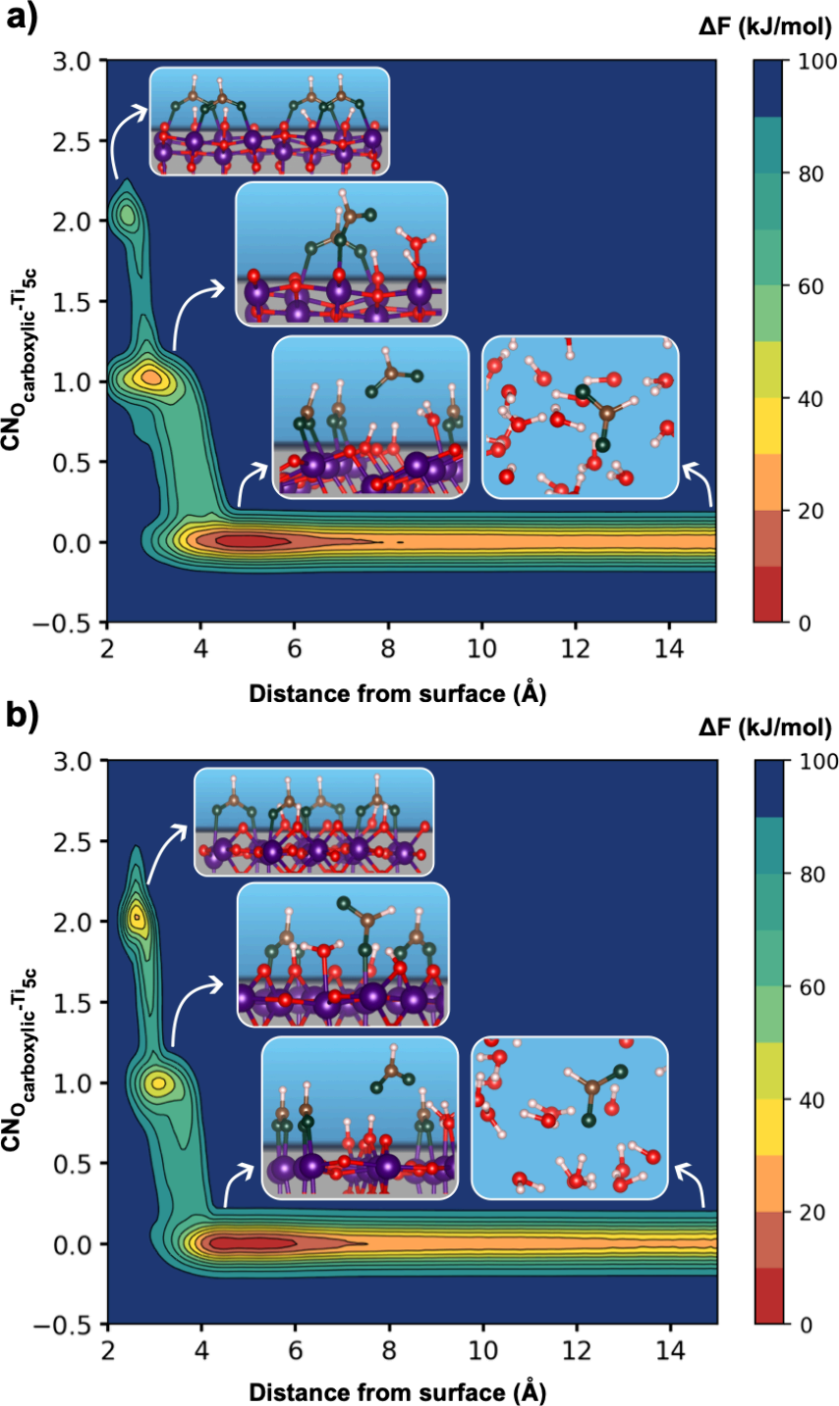
FES for the adsorption/desorption of a single FA from 0.5 ML FA-covered (a) A101 surface and (b) R110 surface. The inset shows the atomic structures of the different states (color codes: indigo, Ti; red, lattice and water O; dark green, carboxylic acid O; brown, C; and white, H). Reproduced with permission from ref 20. Copyright 2025 Wiley-VCH GmbH.

However, the most interesting feature of the FES is the location of the free energy minimum, which corresponds to a state where the FA is localized in the vicinity of the interface (CN_O_carboxylic_–Ti_5c__ ≈ 0, *D* ≈ 4–5 Å), rather than in the bulk water region (CN_O_carboxylic_–Ti_5c__ ≈ 0, *D* > 10 Å). This localized state is characterized by FA that is just desorbed as HCOO^–^ strongly interacting with the acid protons on the O_2c_ sites or adsorbed water on the Ti_5c_ sites. Crucially, the location of the free energy minimum was identical across the FA and AA-terminated A101 and R110 surfaces at different coverages, suggesting a universality in the preference of this localized state, which is ∼10–30 kJ/mol lower in energy than the completely desorbed state. These results thus indicate that, under ambient conditions, atmospheric organic acids such as FA or AA prefer to localize in the interfacial water layers adjacent to the TiO_2_ surface but do not chemisorb by displacing the adsorbed water on the Ti_5c_ sites, a finding that is different from observations made under ultrahigh vacuum (UHV) conditions.^44^

This interfacial localized state was further characterized by considering 0.125 ML FA and AA-covered A101 and R110 surfaces interfaced with a 0.36 M FA or AA solution. Over nanosecond-time-scale simulations, we found that the FA in solution spontaneously localized at the interface but did not displace the adsorbed water on the Ti_5c_ sites. A similar behavior was observed for AA, though with a lower propensity for localization. For FA, this localization was found to be driven by a strong interaction between the acid proton and the surface O_2c_ sites through a proton transfer process. Specifically, the FA in solution approaches the interfacial water layers and spontaneously transfers its acid proton to the surface O_2c_ sites, forming a HCOO^–^ and O_2c_H^+^ ion pair that stabilizes the localized state. On the other hand, this is seldom observed in the case of AA, which prefers to remain as CH_3_COOH. Taken together, this suggests that the key factor determining the localization of atmospheric carboxylic acids near aqueous TiO_2_ interfaces is acid–base chemistry dictated by a combination of the p*K*_a_ of the acid and the pH_PZC_ of the surface.

To understand the influence of these acids on the hydrophobicity of TiO_2_ in the dark, we further characterized the propensity of water to dewet the surface. Both theory and simulations have established that enhanced water depletion near solutes and surfaces can be considered as the molecular fingerprint of hydrophobicity.^47,48^ In practice, this is estimated through the fluctuations in water density within a probe volume near the interface. Specifically, the larger the probability of observing *N* = 0 water molecules, i.e., the larger the value of *P*(*N* = 0), the easier it is to dewet the surface, making it more hydrophobic. Figure 4a,b shows the probability distributions of water density fluctuations in a spherical probe volume of radius 3.3 Å for the different FA-covered/localized A101 and R110 water interfaces, respectively. The probe volume was placed both near the interface and in the bulk water region to estimate the water density fluctuations.

**Figure 4:**
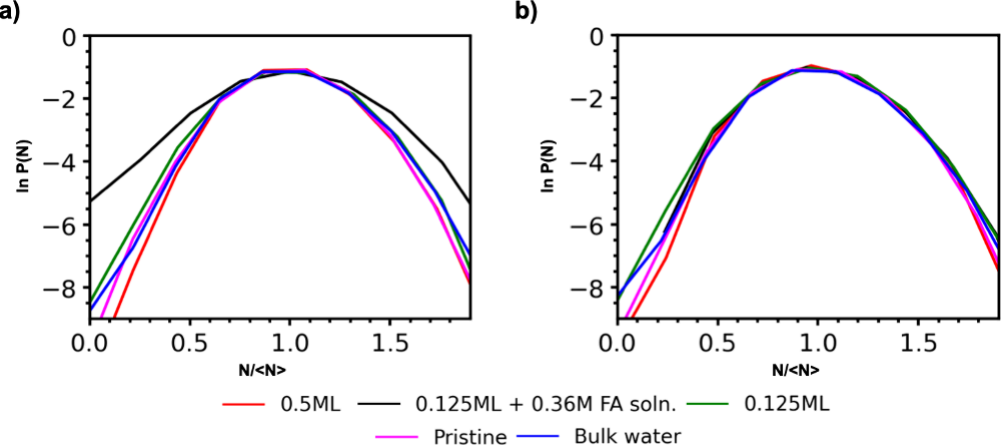
Water density fluctuations recorded in a spherical probe volume near the interface and in the bulk water region for different FA-covered/localized (a) A101–water and (b) R110–water interfaces. The signature for hydrophobicity is characterized by an enhanced probability to observe zero water molecules within the probe volume, i.e., *P*(*N* = 0). Reproduced with permission from ref 20. Copyright 2025 Wiley-VCH GmbH.

It can be seen that only the FA-localized A101–water interface shows enhanced *P*(*N* = 0), with an order of magnitude higher propensity to deplete water near the interface. Instead, the fully FA-covered A101 and R110 interfaces do not show any signature of enhanced water density fluctuations, suggesting that the chemisorbed monolayer of carboxylic acids observed under UHV conditions is unlikely to be the cause of the hydrophobicity of TiO_2_ surfaces in the dark.^44^ Moreover, despite the observed localization of FA near the R110 surface, there is no signature of hydrophobicity. This suggests that the larger fraction of dissociated water on the R110 surface (∼22%)^12^ compared to that on the A101 surface (∼6%)^10^ plays a role in reducing the hydrophobicity. On the other hand, all the AA-covered surfaces, regardless of coverage, show enhanced water density fluctuations, making them hydrophobic. In this scenario, the large nonpolar CH_3_ group plays a prominent role in controlling the wettability of TiO_2_. However, given the larger atmospheric abundance of FA over AA,^44^ it can be hypothesized that it is the localized FA, controlled by the complex acid–base chemistry of the interface, that contributes the most to the hydrophobicity of TiO_2_ surfaces in the dark.

## 5 Methanol at TiO_2_–Water Interfaces and How It Affects Water Dissociation

Methanol is known to promote photocatalytic H_2_ production on TiO_2_ surfaces either by acting as a hole scavenger to inhibit the recombination of photoexcited electron–hole pairs or by direct methanol photoreforming.^49,50^ The prevailing mechanistic picture of photocatalytic H_2_ evolution on TiO_2_ holds that adsorbed methanol and water at Ti_5c_ sites first dissociate to form terminal methoxyls and terminal hydroxyls (OH_t_), respectively, together with bridging hydroxyls (OH_br_).^51^ Subsequently, OH_br_ accepts electrons to evolve H_2_, while terminal methoxyls or terminal hydroxyls accept holes and are oxidized to formaldehyde or hydroxyl radicals, respectively.^52–54^ Because the oxygen evolution reaction (OER) is widely regarded as the primary bottleneck in photocatalytic water splitting, replacing water oxidation with methanol oxidation tends to promote H_2_ production.

However, questions remain regarding the detailed mechanisms of these processes at TiO_2_–water interfaces where water and methanol are coadsorbed. Previous studies under UHV conditions have provided valuable insights into the adsorption mode and photo-oxidation pathways of methanol at TiO_2_–vacuum interfaces.^55,56^ Under such conditions, methanol is usually reported to adsorb molecularly on anatase, whereas thermal dissociation into surface methoxyls and bridging hydroxyls is prevalent on rutile.^57,58^ In addition, only surface methoxyl groups are found to be photoactive and capable of scavenging photogenerated holes, indicating that methanol must first dissociate to induce enhancement in photocatalytic H_2_ production.^56,59^ These results suggest that it is important to identify the dissociation dynamics of both water and methanol coadsorbed at TiO_2_–water interfaces in order to elucidate the microscopic mechanism by which methanol modulates H_2_ production.

To address these questions, we employed a DP trained on a dataset comprising anatase TiO_2_(101)–water and rutile TiO_2_(110)–water interfaces modified with adsorbed methanol on the oxide surfaces, with reference forces labeled using the meta-GGA SCAN functional.^27^ Using these DPs, we performed 10 ns DPMD simulations of the anatase and rutile TiO_2_–water interfaces modified with 0.5 ML adsorbed methanol, complemented by enhanced sampling simulations to quantitatively determine the free energy profiles associated with all dissociation reactions.^21^ To this end, we performed well-tempered metadynamics simulations^35,40^ using three collective variables (CVs) that track the numbers of formed OH_br_ species (i.e., the O_2c_ coordination number), dissociated methanol, and dissociated water (details on the CVs are given in the SI). Convergence of the metadynamics simulations was verified via the diffusive behavior of the CVs (see Figure S8 in the SI). The resulting free energy surfaces were projected onto each CV to obtain one-dimensional free energy profiles, shown in Figure 5 for both anatase and rutile surfaces at methanol coverages of 0.125 and 0.5 ML. At 0.125 ML coverage, both phases exhibit behaviors essentially identical to those of the bare TiO_2_–water interfaces. Under these conditions, anatase seldom undergoes either methanol or water dissociation, as indicated by the monotonic free energy increase with sequential dissociation. When the anatase surface is modified with 0.5 ML adsorbed methanol, all dissociation reactions become more favorable, reflected by a systematic downward shift of the free energy curves. These trends are quantified by the Boltzmann-averaged dissociation fractions reported in Table 1, which show minuscule water and methanol dissociation fractions on anatase with 0.125 ML adsorbed methanol and a substantial increase in all dissociation fractions at 0.5 ML coverage. We also note that the free energy trends remain consistent regardless of the projection scheme, as observed in the 2D projection presented in the SI.

**Table 1:** Average Fractions of Methanol and Water Dissociation and O_2c_ Hydroxylation on the Anatase TiO_2_(101) and Rutile TiO_2_(110) Surfaces Obtained from the Calculated Free Energies

Surface Methanol Coverage	Methanol Dissociation	O_2c_ Hydroxylation	Water Dissociation
Anatase-0.125 ML	4.3%	3.4%	3.2%
Anatase-0.5 ML	12.3%	14.1%	15.0%
Rutile-0.125 ML	39.7%	24.3%	21.8%
Rutile-0.5 ML	47.5%	24.5%	1.2%

**Figure 5:**
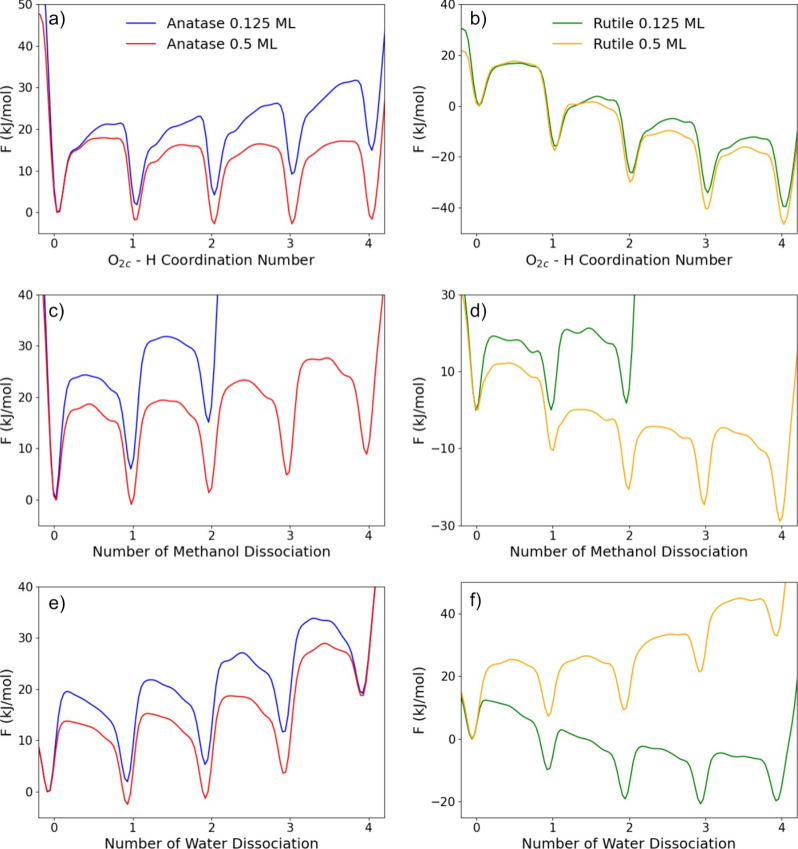
One-dimensional free energy profiles for O_2c_ hydroxylation (a, b), methanol dissociation (c, d), and water dissociation (e, f). On the anatase (101) surface, 0.5 ML methanol adsorption lowers the free energies of all dissociation processes in comparison to 0.125 ML methanol, indicating an increased population of surface dissociated species. On rutile (110), methanol dissociation is favored, whereas water dissociation is strongly suppressed. In contrast, the O_2c_ hydroxylation free energy profile remains largely insensitive to methanol coverage. The numbers of methanol and water dissociation are calculated from the O_me_–H and O_w_–H coordination numbers, respectively. Adapted from ref 21. Copyright 2025 American Chemical Society.

In contrast to anatase, rutile at 0.125 ML methanol coverage exhibits both prominent water and methanol dissociation, reflected by the relatively low free energies of states with increasing numbers of dissociated water molecules. This behavior is also evident from the large water dissociation fraction, with more than 20% of adsorbed water remaining dissociated. Most interestingly, an unexpected trend emerges at 0.5 ML coverage on rutile: water dissociation becomes unfavorable, while methanol dissociation is strongly promoted, as indicated by an almost vanishing water dissociation fraction and a methanol dissociation fraction of *∼*50%. Overall, the effect of 0.5 ML methanol adsorption turns out to be strikingly different between anatase and rutile. While both water and methanol dissociation are enhanced on anatase, the prevalent water dissociation on rutile is effectively replaced by methanol dissociation.

To gain insight into the different behaviors of anatase and rutile in response to methanol adsorption, we performed a detailed analysis of the DPMD trajectories, which revealed that terminal methoxyl groups can mediate and facilitate water dissociation on anatase. This mechanism is illustrated in Figure 6a: methanol first dissociates (**1** → **2**), after which the resulting methoxyl group abstracts a proton from an adjacent water molecule (**2** → **3**), ultimately inducing water dissociation. Similar reactions have been discussed in previous experimental studies, often referred to as the indirect oxidation pathway, in which redox reactions occur across adsorbates rather than directly from TiO_2_.^49,51^ To quantify the free energies associated with these two reaction steps under aqueous conditions, we performed umbrella sampling calculations,^35,60^ with the resulting free energy profiles shown in Figure 6b,c. The initial methanol dissociation step is thermodynamically disfavored on anatase, whereas dissociated methanol is relatively stable on rutile. This trend is reversed for the second reaction step, in which methoxyl-mediated water dissociation is favorable on anatase but unfavorable on rutile.

**Figure 6:**
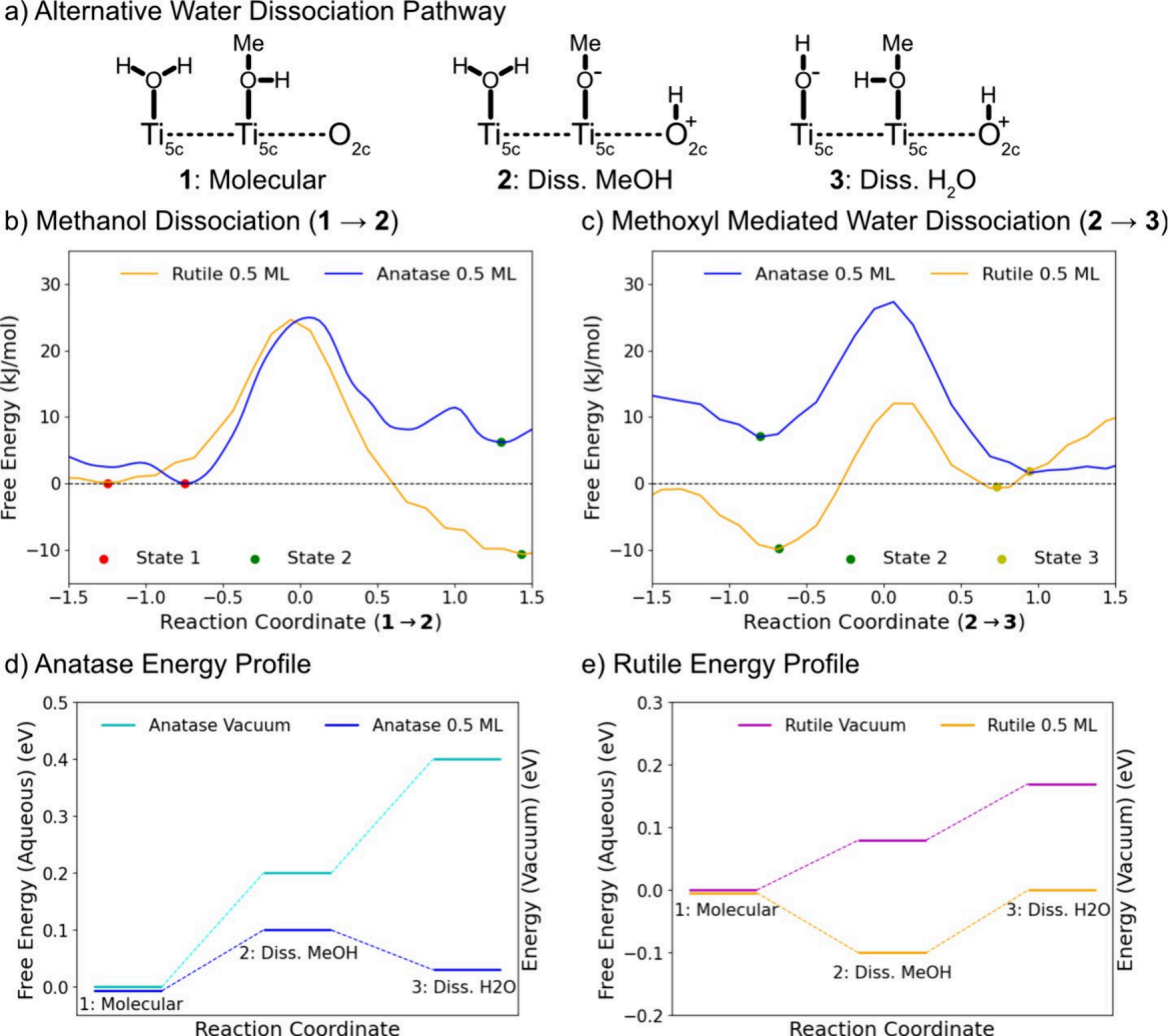
Initial (**1**), intermediate (**2**), and final (**3**) states of the methanol-mediated water dissociation pathway. The overall process consists of two steps: initial methanol dissociation (**1** → **2**) followed by methoxyl-mediated water dissociation (**2** → **3**). (b, c) Free energy profiles of the first (left) and second (right) reaction steps for anatase (blue) and rutile (orange) interfaces along the corresponding reaction coordinates. While the first reaction step is favored on rutile, the second is favored on anatase. Red, green, and yellow dots denote states **1**, **2**, and **3**, respectively, with the zero of free energy set at state **1**. (d, e) Relative free energies of states **1**, **2**, and **3** at the vacuum (aqueous) anatase (left) and rutile (right) interfaces. Adapted from ref 21. Copyright 2025 American Chemical Society.

These results are summarized in Figure 6d,e, which show the relative free energies of states **1**, **2**, and **3** at the aqueous anatase and rutile interfaces, as well as at the corresponding vacuum interfaces with one methanol and one water molecule coadsorbed on the surface. Clearly, both water and methanol dissociation are energetically uphill in vacuum since the formation of charge-separated species is unfavorable in the absence of solvent stabilization. Interestingly, state **3** exhibits much stronger solvent stabilization on anatase, which can be attributed to the inherent surface structure with longer Ti_5c_–Ti_5c_ distances than those of rutile. This allows water molecules to approach the surface more closely and form a more favorable hydrogen bonding environment around the charged OH_t_ species.

Overall, the free energy profiles of the two reactions clarify the origins of the markedly different behaviors of the two TiO_2_ phases under methanol adsorption. On anatase, methanol dissociation turns out to be thermodynamically unfavorable, as it rapidly returns to the molecular state by abstracting a proton from either OH_br_ species or adsorbed water, thereby introducing an additional pathway for water dissociation. In contrast, methanol dissociation is strongly favored on rutile, even more so than water dissociation, effectively suppressing the latter. These findings have significant implications for the photocatalytic H_2_ evolution pathways on the two TiO_2_ phases. On anatase, the increased population of photoactive dissociated species is expected to enhance photocatalytic activity along with the concurrent consumption of both water and methanol. On rutile, instead, methanol dissociation effectively replaces water dissociation, and photocatalytic enhancement is anticipated to arise from the use of methanol as a more efficient hole scavenger, ultimately suppressing electron–hole recombination. These observations are consistent with available experimental evidence, although further verification is warranted.

## 6 Concluding Remarks

Advanced molecular simulations enabled by MLPs, often coupled with enhanced sampling methods, have helped usher in a new paradigm of modeling complex aqueous oxide interfaces of relevance to several contemporary applications. To illustrate these capabilities, in this Account we have discussed how the acid–base chemistry of IrO_2_ can contribute to its OER activity, how organic acids at TiO_2_–water interfaces modulate surface hydrophobicity, and how methanol adsorption on TiO_2_ promotes H_2_ production in photocatalytic water splitting. However, the sample set of systems discussed in this Account is just the tip of the iceberg in both the potential and the opportunities that lie ahead in utilizing MLPs to understand, optimize, and control complex phenomena in similar systems. The continuous improvements in machine learning algorithms and the ever-increasing computational power at our disposal are already starting to push the frontiers of simulations, enabling access to previously unthinkable length and time scales. However, physical grounding through detailed validation against first-principles methods or experimentally accessible data should be enforced to improve the trustworthiness of a developed MLP. The future thus holds great promise for synergistic computational and experimental approaches to reveal the fascinating phenomena at aqueous interfaces that still remain beyond the reach of current methods.

## Supplementary Material



## Data Availability

All the data sets pertaining to this work are available on public repositories. In particular, the data set for the IrO_2_–water interface is available in the Figshare database and can be accessed using the following links: 10.6084/m9.figshare.28648610 (raw files); 10.6084/m9.figshare.28648643 (training input); and 10.6084/m9.figshare.28648586 (frozen models). The training data set, input files used for training, and deep potential models generated in the study on the adsorption of carboxylic acids at TiO_2_–water interfaces are available in the Figshare database and can be accessed using the following link: https://figshare.com/s/308898427f80dc098f7d. All data sets generated in the study on methanol at TiO_2_–water interfaces are available at https://github.com/sanghyunjonathan/Methanol-Adsorbed-TiO2-Interface.

## Notes

Detailed information on the training and validation of the deep potentials and on the enhanced sampling simulations for each of the three aqueous oxide interfaces discussed in this Account (PDF)
