# Supplementary material for: Understanding Adsorption and Reactions at Aqueous Oxide Interfaces with Neural Network Potential Molecular Dynamics
Source: Acc Mater Res. 2026 May 14;7(7):699–709. doi: 10.1021/accountsmr.6c00001 (PMC13411064; doi:10.1021/accountsmr.6c00001)
Supplement: Supplementary file 1 [file mr6c00001_si_001.pdf]

Supporting Information for:

Understanding adsorption and reactions at aqueous oxide interfaces with neural network potential molecular dynamics

Sanghyun J. Park,† Abhinav S. Raman,‡ and Annabella Selloni∗,†

†*Department of Chemistry, Princeton University, Princeton, NJ, 08544, USA*
‡*Department of Chemical Engineering, Indian Institute of Technology Madras, Chennai, 600036, India*

E-mail: aselloni@princeton.edu

ADDITIONAL INFORMATION ON THE METHODOLOGY

1. Acid-base chemistry of the IrO_2 – water interface

1.1. Training and validation of the DP

The initial coarse DP of the IrO_2(110)-water interface was obtained by considering ≈100 equally spaced snapshots from an AIMD simulation of a periodic (2 x 4) IrO_2 slab interfaced with a ≈ 16 Å thick water layer between consecutive slabs, such that the bulk water density was 1g/cm^3. This initial coarse DP was used to start the iterative active learning scheme of Zhang et al.[1] In the exploration step of active learning, the following systems and thermodynamic conditions were sampled: a) bulk IrO_2 (96 atoms) at 200-1000 K and 1 bar; b) rutile IrO_2(110)-water interface (432 atoms) at 200-800 K and constant volume; c) water adsorbed on IrO_2(110) surface in vacuum (198 atoms) at 200-600 K and constant volume; and d) bulk water (192 atoms) at configurations taken from Ref.[2] Once the DP was considered converged (average deviation in atomic forces between an ensemble of three DP's < 0.05 eV/Å), it was further refined with configurations involving proton transfer between the different acid-base sites. These configurations were specifically obtained through enhanced sampling simulations described later. The final converged dataset used for training and its compositions are provided in Table S1. For each system, a random subset of configurations from the training set was used as the validation set during training.[3]

Table S1. The complete list of configurations making up the training dataset for the rutile IrO_2(110)-water interface.[3] *Taken from Ref.[2]

System	#atoms	#frames (training)	#frames (validation)
Bulk IrO_2	96	712	100
Water adsorbed on rutile IrO_2 (110) surface in vacuum	198	215	50
Bulk water	192	1599*	80
Rutile IrO_2(110)-water interface	432	7204	180

Initial validation of the DP was performed through a parity plot of the norm of the atomic forces for 100 random configurations of each of the sub-systems making up the training dataset, as shown in Figure S1. Root-mean-square errors (RMSE) are in the range of 130-180 meV/Å, which is similar to other DP models for aqueous oxide interfaces.[2, 4] Additionally, parity plots of the energy and norm of atomic forces were also computed for 100 random configurations involving a free hydronium ion (H_3O^+) in solution after proton transfer at distances $\leq$ 6 Å and $>$ 6 Å; where 6 Å is the cutoff radius used to define the local atomic environments when training the DP. These are shown in Figure S2, having similar RMSE as the global configurations considered in Figure S1, suggesting that the proton

transfer process has been captured accurately, and that the influence of long-range electrostatics is minimal in this system. Additional detailed validation is discussed in Ref.[3]

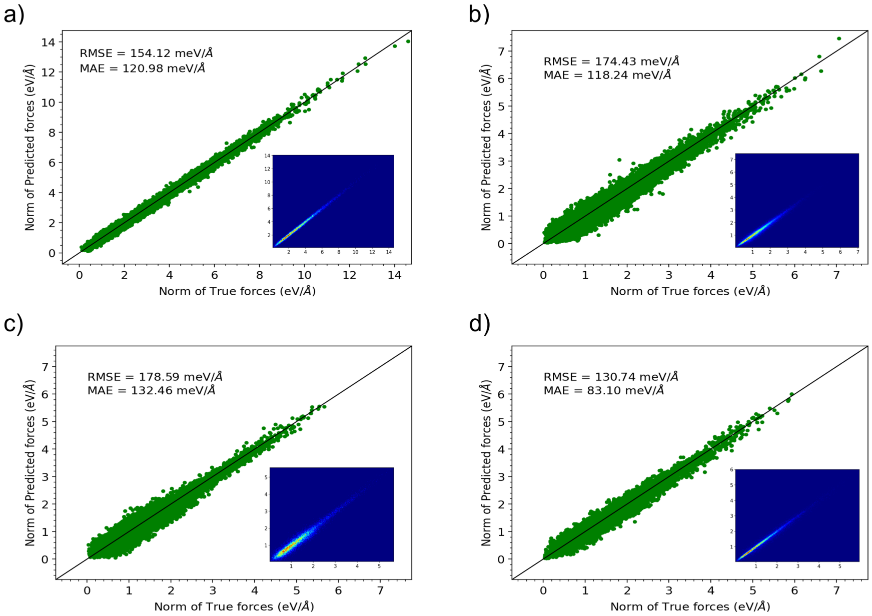

Figure S1: Parity plot of the norm of atomic forces (true: DFT; predicted: DP) for 100 configurations of each of the following systems: a) bulk IrO_2, b) rutile IrO_2(110)-water interface, c) water adsorbed on the rutile IrO_2(110) surface in vacuum, and d) bulk water. Insets show the corresponding 2D-histograms. Reproduced from Ref.[3]

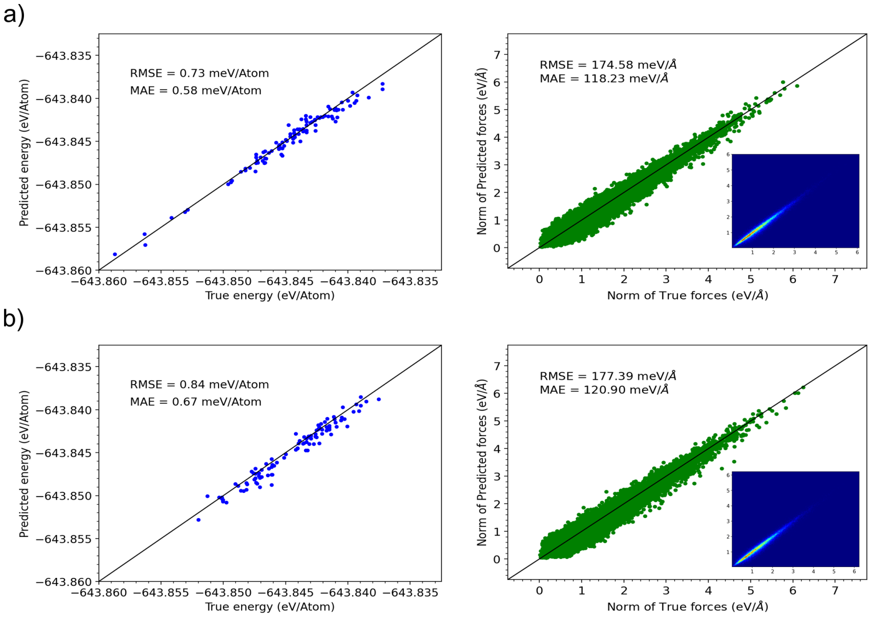

Figure S2: Parity plots showing the energy and atomic forces (true: DFT and predicted: DP; 100 configurations each) for a) configurations involving a free hydronium in solution at distances ≤ 6 Å and b) at distances > 6 Å; where 6 Å is the cut-off radius used to define the atomic environments when training the DP. Insets show the corresponding 2D-histograms. Reproduced from Ref.[3]

1.2 Enhanced Sampling

All enhanced sampling simulations were performed using the enhanced sampling plugin PLUMED.[5] These were used in the DP training and for computing the pH_{PZC} . To model proton transfer, the collective variables (CVs) developed by Grifoni et al.[6] were adapted for this system. Specifically, the protonation state s_p is defined as: $s_p = \sum_{k=0}^{N^s-1} 2^k . q_k$, where k corresponds to the indices used to label the reactive sites, q_k is the total proton excess/defect associated with each reactive species, and N^s is the number of inequivalent reactive sites. The distance between the formed conjugate acid-base pair was estimated using the s_d CV, which is defined as: $s_d = \sum_{i,m>i} -r_{im}. \delta_i . \delta_m$, where the indices run over acid-base sites of the inequivalent groups, r_{im} is the distance between the two sites, and δ_i is the partial charge on the reactive sites. This ensures that only the acid-base pair that has exchanged a proton has a contribution different from zero. Finally, a restraint (s_r) was applied to avoid the formation of more than one conjugate acid-base pair, which is needed for the appropriate use of the CVs. The enhanced sampling simulations were all performed by considering a fully hydroxylated surface (i.e. $OH_{cus}^- = OH_{br}^+$), which served as a standard reference for estimating the proton excess/defect used in the definition of the CVs. To further avoid the rapid transfer of protons between Ir_{cus} and O_{br} which was observed in the equilibrium sampling simulations, an additional restraint on the protonation state of the site that is not being biased to exchange protons was applied. This ensured that the transfer of protons was only between the selected surface sites (cus or br) and water, which is essential for the estimation of the pK_a of that surface site. For the estimation of the pH_{PZC}, well-tempered metadynamics[7] simulations (0.5 ns) was performed using the described setup with each of the three independent DPs that only differed in their initialization. The evolution of the s_p CV is shown in Figure S3, where it is diffusive within the simulation timescale, suggesting effective sampling. The converged free energy surface (FES) was then obtained by averaging over these independent simulations using the method developed in Ref.[8] The complete list of parameters used in the well-tempered metadynamics simulations is given in Table S2.

a) b)

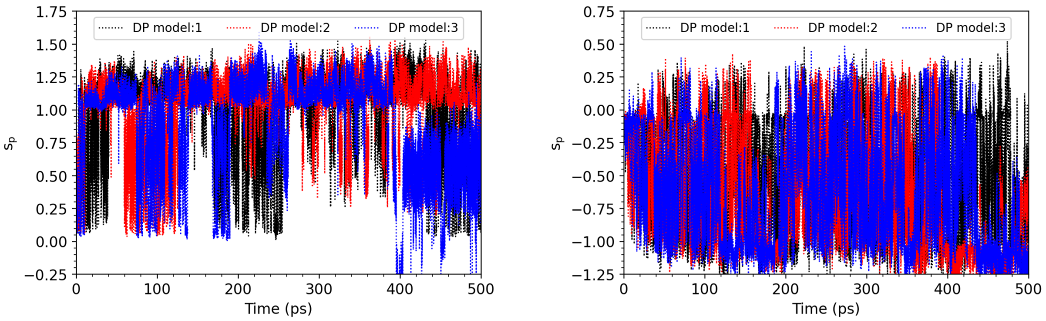

Figure S3: Evolution of the s_p CV for a) the O_{br} site and b) the Ir_{cus} site through the course of the well-tempered metadynamics simulations performed with three different DPs. Reproduced from Ref.[3]

Table S2. Parameters used in the well-tempered metadynamics simulation to evaluate pH_{PZC}. Reproduced from Ref.[3]

Parameter	Value
Gaussian hill height	0.25
Gaussian hill width (s_p)	0.2
Gaussian hill width (s_d)	0.4
Bias factor	10
Hill deposition rate	100
λ (s_p)	7
λ (s_d)	10
λ (s_r)	12
α (s_r)	0.0001

While the free energy of the protonation/deprotonation reactions readily provides estimates of the pK_a, the pH_{PZC} also depends on the value of pK_W, which should be computed based on the auto-ionization of DP water. In this work, we instead considered a sensitivity analysis on the pH_{PZC} and dissociation free energy of adsorbed water (ΔF_{diss}) by providing a range of $\pm 0.5 pK_a$ units relative to the experimental pK_W of 14.0. This is listed in Table S3.[3]

Table S3. Computed deprotonation/protonation free energy (ΔF) and pK_a of the different IrO_2(110) surface sites, pH_{PZC} and ΔF_{diss} obtained from the enhanced sampling simulations, including the performed sensitivity analysis of pK_W. Reproduced from Ref.[3]

Surface site	ΔF (kJ/mol)	pK_a	pH_{PZC}	ΔF_{diss} (kJ/mol)
$OH_{br}^{+} \rightarrow H_3O^{+}$	20.402 $\pm$ 2.293	3.552 $\pm$ 0.399		
			$\boldsymbol{pK_W} = \mathbf{14}$	
$OH_{cus}^{-} \rightarrow H_2O_{cus}$	61.850 $\pm$ 5.189	3.233 $\pm$ 0.903	3.392 $\pm$ 0.552	-1.831 $\pm$ 4.914
			$\boldsymbol{pK_W} = \mathbf{13.5}$	
		2.733 $\pm$ 0.903	3.142 $\pm$ 0.552	-4.703 $\pm$ 4.914
			$\boldsymbol{pK_W} = \mathbf{14.5}$	
		3.733 $\pm$ 0.903	3.642 $\pm$ 0.552	1.41 $\pm$ 4.914

2. Adsorption of organic acids at TiO_2-water interfaces and their effect on the surface hydrophobicity

2.1. Training and validation of the DP

The initial coarse DP was trained using the configurations of formic acid (FA) and acetic acid (AA) covered Anatase TiO_2(101)-A101 and rutile TiO_2(110)-R110-water interface obtained from short AIMD simulations of $\approx$2-3 ps, together with the configurations of the pristine A101 and R110-water interface, bulk rutile and anatase, bulk water, and FA and AA solutions taken from our previous work[2, 4, 9]. The following systems and configurations were considered in the AIMD simulations: For the FA/AA covered A101-water interfaces with the FA/AA adsorbed in the bidentate (BD) configuration at 0.5 ML and 0.125 ML coverage, a symmetric (4 x 1) surface composed of 4 layers interfaced with a 15 Å thick water slab was used. For the mixed (monodentate (MD/BD; 0.67 ML) configuration, a symmetric (3 x 1) surface composed of 4 layers interfaced with an 18 Å thick water slab was used. For all the FA/AA covered R110-water interfaces, a symmetric p (4 x 2) surface composed of 4 layers interfaced with a 15 Å thick water slab was used. The water density was fixed to 1 g/cm^3 in all the cases. Once the initial coarse DP was obtained, the same active learning protocol[1] was employed, where the exploration step sampled configurations corresponding to 200-800 K and constant volume for all the systems. Additionally, enhanced sampling using well-tempered metadynamics[5, 10] was employed to sample configurations involving the desorption/adsorption of FA and AA at the TiO_2-water interface. The complete list of configurations making up the final converged dataset (average deviation in atomic forces between an ensemble of three DP's < 0.05 eV/Å) is listed in Table S4. For each system, a random subset of configurations from the training set was used as the validation set during training.[11]

Table S4. The complete list of configurations making up the training dataset for the FA/AA modified TiO_2-water interface[11]. *Taken from Ref.[2], #Taken from Ref.[9] , and †Taken from Ref.[4]

System	#atoms	#frames (training)	#frames (validation)
Bulk Anatase TiO_2	108	769*	100
A101-water interface	426	13101*	300
Bulk water	192	5583*	200
FA solution (dimers)	382	1240#	100
FA solution (monomers)	98	5758#	200
AA solution (dimers)	388	1740#	100
AA solution (monomers)	101	5845#	200
FA covered A101-water interface (0.5 ML; BD)	466	806	50
FA covered A101-water interface (0.125 ML; BD)	427	1061	50
FA covered A101-water interface (0.67 ML; MD/BD)	358	1762	50
A101-FA solution interface	415	769	50
AA covered A101-water interface (0.5 ML; BD)	490	737	50

AA covered A101-water interface (0.125 ML; BD)	433	984	50
AA covered A101-water interface (0.67 ML; MD/BD)	382	1825	50
A101-AA solution interface	421	658	50
Bulk Rutile TiO_2	216	400†	50
R110-water interface	423	3173†	50
FA covered R110-water interface (0.5 ML; BD)	415	914	50
FA covered R110-water interface (0.125 ML; BD)	421	529	50
AA covered R110-water interface (0.5 ML; BD)	439	1386	50
AA covered R110-water interface (0.125 ML; BD)	427	738	50

Initial validation of the converged DP was performed by computing a parity plot of the norm of atomic forces of the FA/AA covered A101-water interface with both the MD/BD configurations (Figure S4a) and the FA/AA covered R110-water interface in the BD configuration (Figure S4b). For more detailed validation, the reader is referred to Refs.[11, 12].

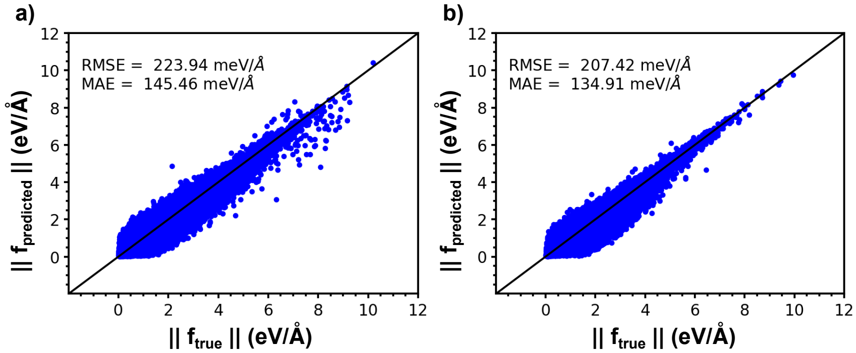

Figure S4: Parity plot of the norm of atomic forces (true: DFT; predicted: DP) for (a) FA/AA covered A101-water interface with both the MD/BD configurations, and (b) FA/AA covered R110-water interface in the BD configuration. Reproduced from Ref.[11]

2.2. Enhanced sampling

Enhanced sampling simulations through well-tempered metadynamics[5] coupled with DPMD simulations were performed (20 ns) to obtain the FES of desorption/adsorption of FA and AA from both the A101 and R110-water interfaces. Three CVs were used to bias the system: i) the z-distance (D) between the surface Ti_{5c} site and the FA/AA carbon ($C_{carboxylic}$), ii) the coordination number (CN) between the surface Ti_{5c} sites and the FA/AA oxygen ($O_{carboxylic}$), and iii) the CN between the surface Ti_{5c} sites and water oxygen (O_W). A continuous coordination number was used based on the following switching function:

$$S_{ij} = \frac{1-\left(\frac{r_{ij}-d_0}{r_0}\right)^n}{1-\left(\frac{r_{ij}-d_0}{r_0}\right)^m} \qquad \text{(S1)}$$

where, r_0 was fixed to 0.75, and d_0 was taken as the position of the first-peak of the $Ti_{5c} - O_W$ or $Ti_{5c} - O_{carboxylic}$ pair correlation function, which was found to be 2.175 Å. Two restraints were used: i) on the CN between Ti_{5c} and $O_{carboxylic}$ such that only one acid desorbed at a time, and ii) on the z-distance such that the desorbed FA/AA do not drift beyond half the box length along the z-direction to avoid any spurious interactions with the periodic interface. The complete list of parameters used in the well-tempered metadynamics simulations are listed in Table S5.

Table S5. Parameters used in the well-tempered metadynamics simulations to evaluate the desorption free energy (ΔF_{des})[11]

Parameter	Value
$CN_{O_{carboxylic}-Ti_{5c}}$	In Eq. S1, $n = 4$, $m = 10$, $d_0 = 2.175$ Å, $r_0 = 0.75$
$CN_{O_W-Ti_{5c}}$	In Eq. S1, $n = 4$, $m = 10$, $d_0 = 2.175$ Å, $r_0 = 0.75$
Restraint on z-distance	κ = 1000 kJ/mol Å^2
Restraint on $CN_{O_{carboxylic}-Ti_{5c}}$	κ = 500 kJ/mol Å^2
Gaussian hill height	2.4942
Gaussian hill width ($D, CN_{O_{carboxylic}-Ti_{5c}}, CN_{O_W-Ti_{5c}}$)	0.1, 0.1, 0.1
Bias factor	10
Hills deposition rate	1000

For all the systems, three independent well-tempered metadynamics simulations coupled with DPMD were performed, each with a DP that only differed in their initialization. The converged unbiased FES was obtained by summing the deposited Gaussian functions that constitute the bias potential. In order to verify the convergence, the ΔF_{des} estimated at 15 and 20ns are reported in Table S6.

Table S6. Free energies of desorption (ΔF_{des}) of FA and AA at the TiO_2-water interface, obtained from well-tempered metadynamics combined with DPMD using three different DPs @15 and 20ns of simulation time. ΔF_{des}, with the corresponding standard deviation, is computed as the free energy difference between the fully desorbed (solvated) state and the acid adsorbed in the BD configuration.[11]

System	ΔF_{des} (kJ/mol) @15ns simulation time		ΔF_{des} (kJ/mol) @20ns simulation time	
	A101	R110	A101	R110
FA (0.5 ML; BD)	-49.4 $\pm$ 10.1	-15.4 $\pm$ 8.0	-44.6 $\pm$ 8.0	-20.1 $\pm$ 9.9
AA (0.5 ML; BD)	-50.0 $\pm$ 7.8	-31.9 $\pm$ 11.1	-55.4 $\pm$ 11.6	-38.9 $\pm$ 14.7
FA (0.125 ML; BD)	-53.5 $\pm$ 8.4	-39.5 $\pm$ 6.0	-58.1 $\pm$ 11.0	-43.0 $\pm$ 7.8
AA (0.125 ML; BD)	-55.9 $\pm$ 1.3	-31.4 $\pm$ 10.8	-60.4 $\pm$ 7.3	-35.0 $\pm$ 15.2

3. Methanol at TiO_2-water interfaces and how it affects water dissociation

3.1. Training and validation of the DP

For training and active learning, we included configurations from the following systems: four-layer anatase (101) - (1×4) and rutile (110) - (2×4) slabs, each terminated by a low (0.125 ML) or high (0.5 ML) coverage of adsorbed methanol and interfaced with a 15 Å slab of water or aqueous methanol solution. The water density was fixed at 1 g/cm^3. In addition, aqueous methanol solutions at two different concentrations were included. To generate an initial training set for these systems, we performed 10 ps AIMD simulations and selected 100 configurations, which were then labeled using the Quantum ESPRESSO[13] package. The initial set consisted of 800 labeled configurations, combined with approximately 23,000 configurations comprising bulk anatase and rutile TiO_2, bulk liquid water, and anatase (101)–water and rutile (110)–water interfaces adopted from previous works[2, 4, 12].

With the initial training set in hand, a coarse DP was trained to initiate the active learning procedure. The DP neural network consisted of a 25 ×50 ×100 embedding nets, followed by a 120 ×120 ×120 fitting net. Training was carried out for 1,000,000 optimization steps with the learning rate decaying from 0.005 to 1.75×10^{-7}. At each active learning iteration, three independent DPs initialized with different random seeds were trained on the same dataset. The exploration step was then performed by running DPMD simulations at temperatures ranging from 200 K to 800 K for up to 100 ps. Subsequently, configurations with maximum force deviations between 0.1 and 0.8 eV/Å were selected for labeling and added to the training set. The active learning procedure was considered converged when

the average force deviation fell below 0.05 eV/Å over a 100 ps-long DPMD trajectory.

To accurately model methanol desorption and proton transfer processes using the trained DP, it is essential to include relevant configurations in the training set. Such configurations were obtained from enhanced sampling simulations specifically designed to explore these reaction pathways (see below). Umbrella sampling[14] was used to sample reactants, products, and transition states of proton transfer reactions, where a proton was gradually driven from methanol to a designated O_{2c} site. Metadynamics simulations[10] employing a surface-to-methanol z-distance collective variable (CV) were used to generate configurations associated with methanol desorption. The final training set consisted of approximately 30,000 configurations, as summarized in Table S7.

Table S7: Configurations in the training set (* Taken from Refs.[2, 4])

System	# of frames
Bulk Water	5583*
Bulk Anatase	769*
Bulk Rutile	400*
Anatase–water	13101*
Rutile–water	3173*
Anatase-0.125ML MeOH	939
Anatase–0.5ML MeOH	1113
Anatase–MeOH solution	529
Rutile–0.125ML MeOH	920
Rutile–0.5ML MeOH	1113
Rutile–MeOH solution	484
MeOH solution	1337

The training set error parity plots for one of the DPs are shown in Figure S5 for the systems under 0.125 ML (low) and 0.5 ML (high) coverage of methanol. The resulting energy and force errors are comparable to those reported in previous studies on TiO_2–water interface systems. The validation set was constructed by sampling configurations from 10 ns equilibrium DPMD trajectories propagated with the 0.125 ML and 0.5 ML systems. Similar parity plots for the validation set using all three DPs are presented in Figure S6. Overall, the validation set errors are comparable to, or perhaps slightly lower than the training set errors,

indicating that the trained DPs are faithful in predicting energies and forces on relevant yet unexplored configurations.

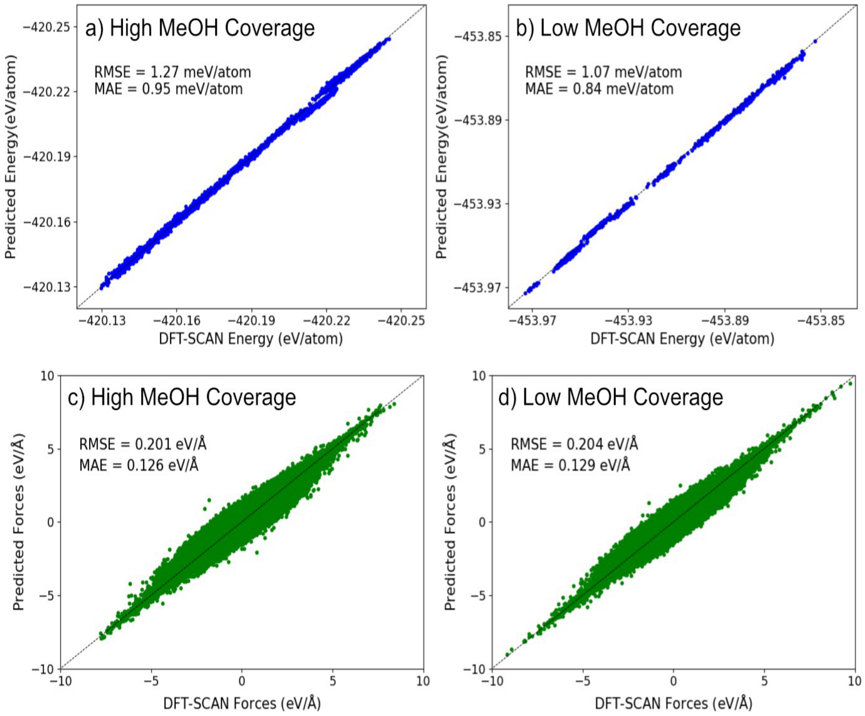

Figure S5: Parity plots of total energies (a,b) and forces (c,d) predicted by the trained DP versus SCAN for high (0.5 ML) and low (0.125 ML) methanol coverage configurations from the **training set**. Each dot in the upper panels represents total energy of a single configuration, while each dot in the lower panels corresponds to either x, y, or z force component on a single atom. Predictions closer to the diagonal line indicate higher DP accuracy. Root mean squared errors (RMSE) and mean absolute errors (MAE) are shown inside each panel. Reproduced from ref.[15]

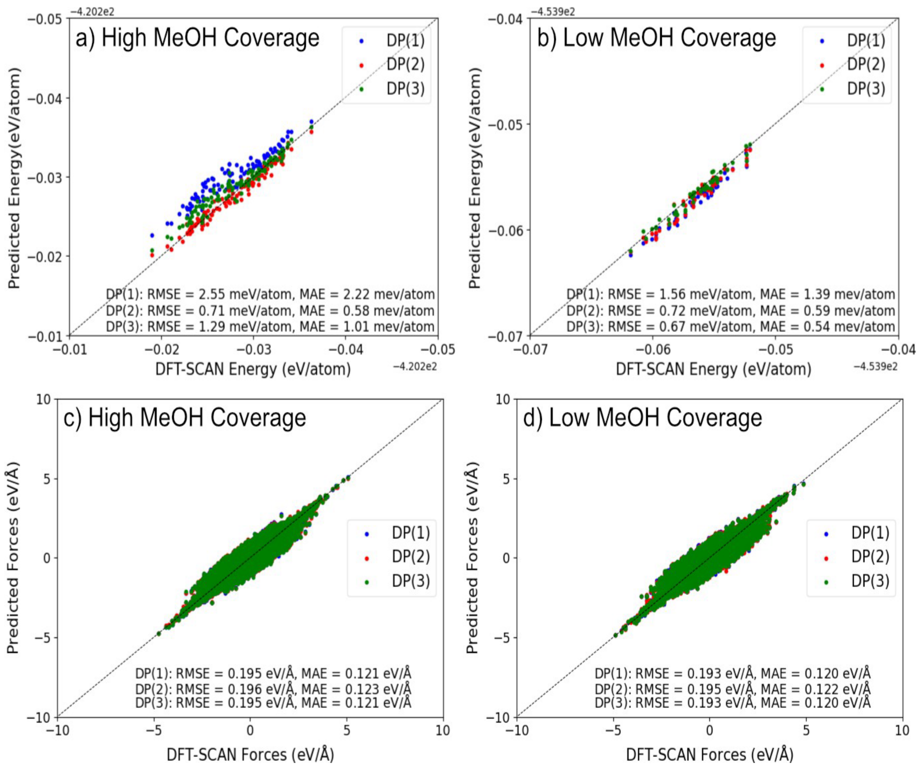

Figure S6: Parity plots of total energies (a,b) and forces (c,d) predicted by all three DPs versus the SCAN functional for high and low coverage TiO_2–water interface configurations from the **validation set.** Reproduced from ref .[15]

3.2 Enhanced Sampling

All enhanced sampling simulations were performed using only one of the three trained DPs. Umbrella sampling calculations were performed to estimate the free energy profile of the direct methanol dissociation and methoxy-mediated water dissociation processes. A collective variable (CV) defined as $d_1 - d_2$ was used, in which d_1 and d_2 correspond to the O_{Me}–H and O_{2c}–H distances or O_w–H and O_{Me}–H distances depending on the reaction. A harmonic potential with a force constant of 500 kJ mol^{-1} $Å^{-2}$ was applied in 25 simulation windows, each assigned a CV value ranging from -2.5 to 2.5 Å. To collect statistics, the DPMD simulations were propagated for at least 200 ps at 300 K in each window where the uncorrelated CV values were sampled. Free energy profiles were constructed using the Weighted Histogram Analysis Method (WHAM), and associated uncertainties were estimated by Monte Carlo bootstrapping.[16] In addition, a series of restraints was applied to suppress unintended processes other than the designated reaction. For direct methanol dissociation as an example, assuming that a single methanol molecule and an O_{2c} site were selected, the following restraints were applied with a force constant of 1000 kJ mol^{-1} $Å^{-2}$:

1. All adsorbed methanol molecules cannot desorb
2. The selected O_{2c} and methanol cannot accept protons from the unselected species
3. The selected proton cannot be transferred to the unselected O_{2c} sites

These restrictions ensure selective sampling of the direct dissociation pathway, enabling an accurate evaluation of its free energy.

To obtain free energy profiles of methanol desorption on both anatase and rutile surfaces, a similar umbrella sampling scheme was employed, using a CV representing the z-distance between a selected surface adsorbed methanol and Ti_{5c} sites.[17] All other methanol molecules were constrained to remain adsorbed throughout the simulation, and dissociation was prohibited for all methanol. A total of 36 simulation windows, each with assigned CV values ranging from 1.7-5.0 Å and force constants between 500-1500 kJ mol^{-1} $Å^{-2}$, were propagated for 200 ps. The unbiased free energy surface was obtained using the Weighted Histogram Analysis Method (WHAM).

To estimate the free energy of concerted methanol dissociation, well-tempered metadynamics simulations were carried out using 2 CVs defined as follows.

$$CV_1 = (d_1 + d_2)/2 \tag{S2}$$

$$CV_2 = (v_1 + v_2)/2 \tag{S3}$$

$$v_i = b_i - h_i \tag{S4}$$

The definitions of *d*, *b*, and *h* are illustrated in Figure S7. Similarly to the aforementioned umbrella sampling, multiple restraints were applied to inhibit irrelevant and subsequent reactions. In short, the restraints were configured so that only the designated proton transfer reaction could occur—H_1 transferring between methanol and water and H_2 between water and O_{2c}—enabling an accurate estimation of the free energy of the concerted methanol dissociation. To prevent oversampling and promote faster convergence, upper and lower walls were applied to both the CV_1 and CV_2. Gaussians were deposited every 1000 timesteps, with a bias factor set to 10 at 300 K. The final free energy surface was reconstructed by summing the Gaussian hills deposited throughout the simulation.

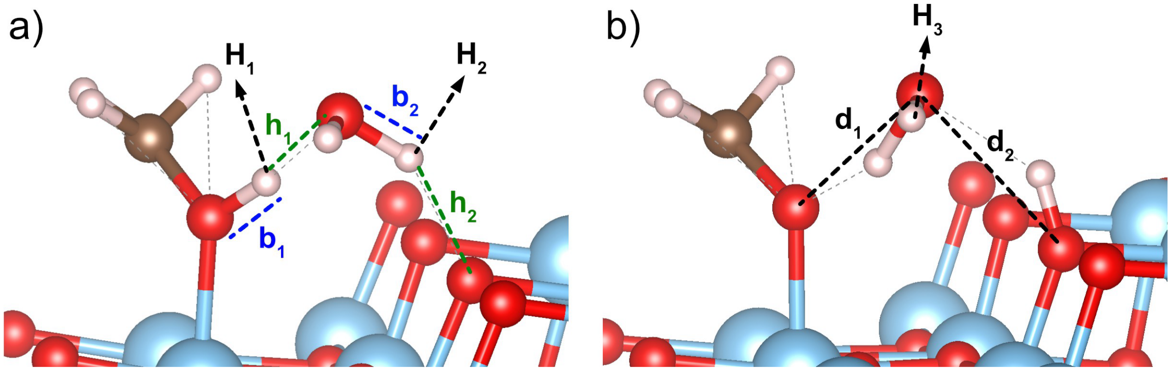

Figure S7: Atomic distances used to define the CVs employed in metadynamics calculations are represented by dashed green, blue, and black lines, corresponding to *h*, *b*, and *d*, respectively. Panels a and b show snapshots before and after the concerted dissociation process. Three hydrogen atoms are labeled; H_1 and H_2 directly participate in the transfer process, while H_3 does not and is constrained to prevent dissociation. Reproduced from ref.[15]

Well-tempered metadynamics simulations were carried out to compute the free energy of water dissociation, methanol dissociation, and O_{2c} hydroxlation using three CVs: coordination numbers of O_{2c}–H, O_{Me}–H, and O_w–H pairs. The coordination number CVs are defined as in Eq. S1. Here, the parameters d_0, r_0, n, and m were chosen for each CN to represent only the first coordination shell, with their values listed in Table S8. Upper and lower bounds of four were applied to the values of the coordination number CVs to prevent oversampling and to promote faster convergence. Additionally, methanol desorption was suppressed to accurately represent both 0.5 ML and 0.125 ML coverage environments. Gaussians were deposited every 1000 timesteps, with a height of 1.5 kJ mol^{-1} and a width of 0.1 CV unit, and a bias factor of 10. All three CVs show dispersive behavior by the end of the simulation, as shown in Figure S8, indicating good convergence in the metadynamics simulations.

The one-dimensional free energy profiles shown in the main text are obtained by Boltzmann integration over all other CVs. Exemplary two-dimensional free energy profiles projected

onto the O_{2c}–H and O_{Me}–H CVs are shown in Figure S9. Here, the profiles show distinct grid-like features favoring integer CV states in the upper diagonal region, indicating that the O_{2c}–H coordination number is determined by changes in the O_{Me}–H and O_w–H CVs, and are therefore constrained by the overall redistribution of protons among surface methanol and water molecules. Additionally, the trends in the one-dimensional free energy profiles are in good agreement with those in the two-dimensional projections.

Table S8: Parameters for coordination number CVs (see Eq. S1).

Coordination Number Pairs	d_0	r_0	n	m
O_{2c}–H	0.8	0.45	12	24
O_{Me}–H	0.8	0.45	12	24
O_w–H	0.8	0.45	12	24
Ti_{5c}–O_{Me}	2.1	0.75	4	10
Ti_{5c}–O_w	2.1	0.75	4	10

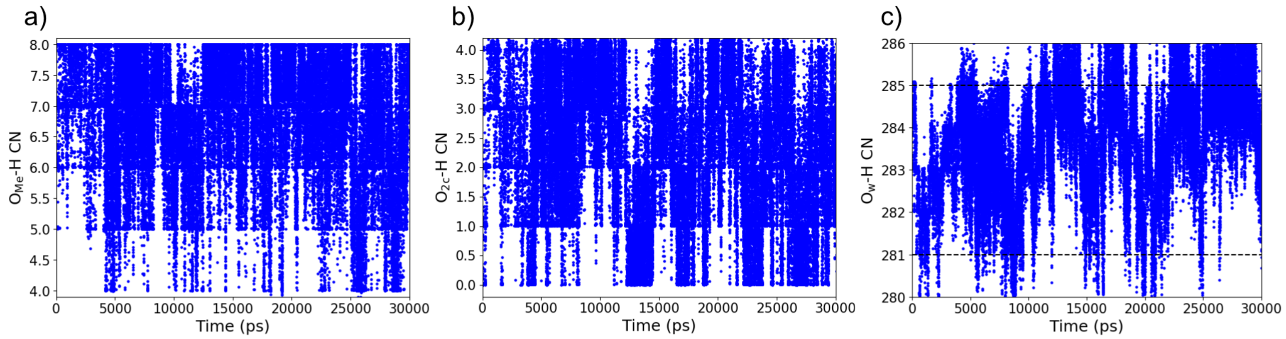

Figure S8: Evolution of a) the O_{me}-H, b) the O_{2c}–H, and c) the O_w-H coordination number CVs over the course of the well-tempered metadynamics simulations for the anatase 0.5 ML system. Reproduced from ref. [15]

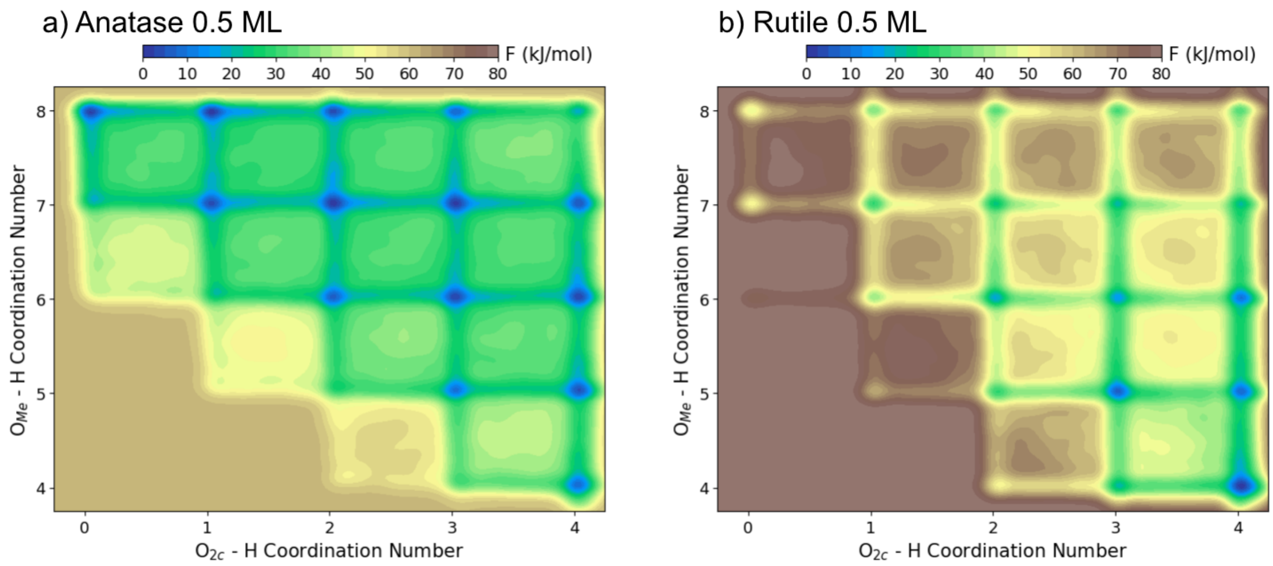

Figure S9: Two-dimensional projections of the free energy profiles from the metadynamics simulations of the 0.5 ML anatase (a) and rutile (b) systems, projected onto the O_{me}-H and O_{2c}-H coordination number CVs. The colors represent free energy values, with darker blue indicating lower free energy. Reproduced from ref. [15]

REFERENCES

(1) Zhang, L.; Lin, D.-Y.; Wang, H.; Car, R.; E, W. Active learning of uniformly accurate interatomic potentials for materials simulation. *Physical Review Materials* **2019**, *3* (2). DOI: 10.1103/PhysRevMaterials.3.023804.
(2) Calegari Andrade, M. F.; Ko, H.-Y.; Zhang, L.; Car, R.; Selloni, A. Free energy of proton transfer at the water–TiO_2 interface from ab initio deep potential molecular dynamics. *Chemical Science* **2020**. DOI: 10.1039/c9sc05116c.
(3) Raman, A. S.; Selloni, A. Acid–Base Chemistry of a Model IrO2 Catalytic Interface. *The Journal of Physical Chemistry Letters* **2023**, *14* (35), 7787–7794. DOI: 10.1021/acs.jpclett.3c02001.
(4) Wen, B.; Calegari Andrade, M. F.; Liu, L.-M.; Selloni, A. Water dissociation at the water rutile TiO2(110) interface from ab-initio-based deep neural network simulations. *Proceedings of the National Academy of Sciences* **2023**, *120* (2), e2212250120. DOI: doi:10.1073/pnas.2212250120.
(5) Tribello, G. A.; Bonomi, M.; Branduardi, D.; Camilloni, C.; Bussi, G. PLUMED 2: New feathers for an old bird. *Computer Physics Communications* **2014**, *185* (2), 604–613. DOI: https://doi.org/10.1016/j.cpc.2013.09.018.
(6) Grifoni, E.; Piccini, G.; Parrinello, M. Microscopic description of acid–base equilibrium. *Proceedings of the National Academy of Sciences* **2019**, *116* (10), 4054–4057. DOI: 10.1073/pnas.1819771116 (accessed 2022/04/24).
(7) Barducci, A.; Bussi, G.; Parrinello, M. Well-Tempered Metadynamics: A Smoothly Converging and Tunable Free-Energy Method. *Physical Review Letters* **2008**, *100* (2), 020603. DOI: 10.1103/PhysRevLett.100.020603.
(8) Tiwary, P.; Parrinello, M. A Time-Independent Free Energy Estimator for Metadynamics. *The Journal of Physical Chemistry B* **2015**, *119* (3), 736–742. DOI: 10.1021/jp504920s.
(9) Raman, A. S.; Selloni, A. Modeling the Solvation and Acidity of Carboxylic Acids Using an Ab Initio Deep Neural Network Potential. *The Journal of Physical Chemistry A* **2022**, *126* (40), 7283–7290. DOI: 10.1021/acs.jpca.2c06252.
(10) Bussi, G.; Branduardi, D. Free-Energy Calculations with Metadynamics: Theory and Practice. *Reviews in Computational Chemistry Volume 28* **2015**, 1–49.
(11) Raman, A. S.; Selloni, A. Effect of Ambient Organic Acids on the Water Structure at ${\rm TiO}_{2}$ Interfaces. *Angewandte Chemie International Edition* **2025**, *64* (33), e202507721. DOI: https://doi.org/10.1002/anie.202507721.
(12) Raman, A. S.; Selloni, A. Long timescale molecular dynamics simulations of carboxylic acid-modified anatase TiO2(101)-water interfaces using ab-initio deep neural network potentials. *Surface Science* **2024**, *750*, 122595. DOI: https://doi.org/10.1016/j.susc.2024.122595.
(13) Giannozzi, P.; Baroni, S.; Bonini, N.; Calandra, M.; Car, R.; Cavazzoni, C.; Ceresoli, D.; Chiarotti, G. L.; Cococcioni, M.; Dabo, I.; et al. QUANTUM ESPRESSO: a modular and open-source software project for quantum simulations of materials. *Journal of Physics: Condensed Matter* **2009**, *21* (39), 395502.

(14) Torrie, G. M.; Valleau, J. P. Nonphysical sampling distributions in Monte Carlo free-energy estimation: Umbrella sampling. *Journal of Computational Physics* **1977**, *23* (2), 187–199. DOI: https://doi.org/10.1016/0021-9991(77)90121-8.
(15) Park, S. J.; Raman, A. S.; Selloni, A. Methanol at Water–TiO2 Interfaces: Free Energies of Water and Methanol Dissociation. *ACS Catalysis* **2025**. DOI: 10.1021/acscatal.5c07506.
(16) Kumar, S.; Rosenberg, J. M.; Bouzida, D.; Swendsen, R. H.; Kollman, P. A. THE weighted histogram analysis method for free-energy calculations on biomolecules. I. The method. *Journal of Computational Chemistry* **1992**, *13* (8), 1011–1021. DOI: https://doi.org/10.1002/jcc.540130812 (accessed 2026/03/24).
(17) Rey, J.; Clabaut, P.; Réocreux, R.; Steinmann, S. N.; Michel, C. Mechanistic Investigation and Free Energies of the Reactive Adsorption of Ethanol at the Alumina/Water Interface. *The Journal of Physical Chemistry C* **2022**, *126* (17), 7446–7455. DOI: 10.1021/acs.jpcc.2c00998.
